# Macronutrient and energy metabolism changes in domestic cats when fed cornstarch, whey protein, and, poultry fat

**DOI:** 10.1017/S0007114525105850

**Published:** 2026-02-14

**Authors:** Sophia A. M. Jantzi, Sanjana F. Anan, Jason Brewer, Cindy Lanman, Dave J. Seymour, Etienne Labussière, Michael A. Steele, Anna K. Shoveller

**Affiliations:** 1 Centre for Nutrition Modelling, Department of Animal Biosciences, University of Guelph, Guelph, ON N1G 2W1, Canada; 2 Royal Canin USA Inc., 500 Fountain Lakes Blvd #100, St Charles, MO 63301, USA; 3 Ruminant Research Centre, Trouw Nutrition R&D, P.O. Box 200, 5830 AE Boxmeer, The Netherlands; 4 INRAE – UMR PEGASE, 16, Le Clos, 35590 Saint-Gilles, France

**Keywords:** Calorimetry, Energy expenditure, Feline, Macronutrient oxidation, Gluconeogenesis, Respiratory quotient, Thermogenesis

## Abstract

There is a lack of knowledge available on how cats adjust their macronutrient partitioning due to the consumption of single-macronutrient meals. The objective of this study was to evaluate consumption of a single meal of ingredients that contained foods of strictly carbohydrates (CHO), fat (FAT) or protein (PRO), on energy expenditure (EE) and macronutrient metabolism in cats. Ten domestic shorthair adult cats (1·9 years; 4·12 kg) were fed 22–24 g of chicken fat (FAT), 56–62 g of whey protein solution (PRO) or 54–56 g of cornstarch solution (CHO) for a single day in a randomised complete block design. Indirect calorimetry was conducted for 24 h post-feeding. Mean average EE over 24 h was highest in cats fed PRO (44 kcal/kg BW) and FAT (43 kcal/kg BW) compared with that in cats fed CHO (42 kcal/kg BW; *P* < 0·01). During 0 to 4 h, cats fed FAT had greater EE (49 kcal/kg BW), suggesting that cats respond to oxidising more dietary fat over protein in the early postprandial stage. Mean 24 h respiratory quotient (RQ) was greatest for cats fed CHO (0·76) followed by PRO (0·75) and FAT (0·74; *P* < 0·05). During 4 to 8 h, the RQ of cats fed PRO was the greatest (0·77), suggesting that cats initially increase gluconeogenesis from amino acids for subsequent glucose oxidation. In comparison to omnivores and herbivores, obligate carnivores have unique responses to single macronutrient intake, where they apparently generate energy from carbohydrate metabolism and rely more on gluconeogenic precursors.

Research regarding cat nutrition and metabolism has specifically focused on how mixed diets and different macronutrient contributions can lead to obesity and Type II diabetes mellitus^(1,2)^. However, information is lacking on how cats adjust their macronutrient oxidation rate and energy expenditure (EE) when only fed a single macronutrient. This knowledge may provide insights into how obligate carnivores utilise nutrients from the consumption of a single meal. Indirect calorimetry is considered the gold standard, minimally invasive methodology that measures the exchange of respiratory gases in a standardised, contained environment. The method allows estimation of EE and macronutrient metabolism (respiratory gas exchange and/or respiratory quotient [RQ]), defined as the ratio of carbon dioxide (CO_2_) production to oxygen (O_2_) consumption^(3)^, which is suited for application to domesticated cats^(4)^. Since cats are relatively small and sedentary in nature, this makes them great candidates for indirect calorimetry using respirometry chambers^(4)^.

Compared with omnivores, obligate carnivores like cats exhibit metabolic idiosyncrasies that may alter macronutrient partitioning and limit or change the metabolic use of protein, carbohydrates and/or fats^(5)^. However, their response to being fed single macronutrients has not been investigated. Studies in humans where individual macronutrients were fed observed average RQ of 0·7, 0·8 and 1·0 for fat, protein and carbohydrate or more specifically dietary glucose, respectively^(3)^. Moreover, dogs who were fed excess glucose also reached an RQ of approximately 1·0, suggesting that healthy dogs can efficiently oxidise glucose^(6)^. A previous study found that cats fed high-fat diets had lower fasted RQ (0·75 ± 0·01) compared with those fed high carbohydrate diets (0·78 ± 0·01)^(7)^. Asaro *et al.* ^(8)^ evaluated the effect of different levels of carbohydrates on feline metabolism over different time intervals during a 24 h period. In that study, a slower time to reach peak EE was observed for cats consuming high carbohydrate diets, compared with those fed diets containing low carbohydrates. A faster peak in EE in cats fed protein may be explained by an increase in gluconeogenesis^(9,10)^. Lester *et al.* ^(1)^ and Gooding *et al.* ^(7)^ suggested that the high EE seen in cats fed high-fat diets was due to increased fat oxidation, where a faster peak in EE was observed earlier in the postprandial period, whereas protein and carbohydrates are used later in the post-absorptive period.

Past studies in cats have evaluated the effects of mixed diets with different concentrations of either carbohydrate, protein or fat on the development of obesity and other health-related conditions through the analysis of EE, RQ and macronutrient oxidation data derived from indirect calorimetry^(7,11)^. Similar methods have been used to characterise energy intake and heat production within other carnivorous models, such as mink^(12)^. However, the impact of singularly fed macronutrients on EE and metabolism in obligate carnivores remains unknown. Therefore, by using indirect calorimetry, we can understand the capacity of cats to oxidise carbohydrate, protein and fat when highly enriched single sources are fed, as well as whether this alters the heat increment of feeding (HIF). We hypothesised that cats fed fat as the single energy source would have lower RQ than those fed protein, which in turn would be lower than those fed carbohydrate. We further hypothesised that cats fed protein and fat as single energy sources would have greater EE than cats fed carbohydrates, and that carbohydrates would result in a lower EE due to the idiosyncrasies in carbohydrate metabolism between obligate carnivores and omnivores. To address these hypotheses, the objective of this study was to assess the effects of consumption of whey protein isolate (protein), cornstarch (carbohydrate) and poultry fat (fat) intake on macronutrient partitioning (RQ) and EE after receiving a single meal.

## Materials and methods

All procedures were evaluated and approved by the Institutional Animal Care and Use Committee at Proctor & Gamble Pet Care and complied with the USDA and AALAC animal care and welfare guidelines. The experimental phase of this study was conducted between May and September of 2010.

### Animals

Ten shorthair domestic cats (*n* 5 neutered males; *n* 5 spayed females) of similar age (1·9 (sd 0·50) years) were used in this study. Previous research assessing the effects on EE in cats fed high and low carbohydrate informed the study design^(13)^. A sample size of *n* 10 was calculated using a power of 0·8 and *α* of 0·05 in G * Power software (Dusseldorf, Germany). All cats were healthy throughout the project, with starting body weights (BW) of 4·12 kg (sd 0·320) and finishing weights of 4·08 (sd 0·330) kg. All cats had a body condition score between 4 and 6 on a nine-point scale^(14)^ and were maintained for optimal body condition. Animals were behaviourally acclimated to the procedures used for indirect calorimetry for the recommended 11 weeks^(15)^. Water was provided *ad libitum* via automatic waterers in the common space or bowls in the chamber.

Cats were housed in the same room with indoor and outdoor access from 07.00 to 15.30 h and indoors only from 15.30 h until the next morning. Beds, scratching posts, toys, perches and cat towers were provided for enrichment. For normal feeding days, cats were fed individually in cages (56 cm × 56 cm × 90 cm) at 07.30 h and 13.00 h. Cats received human interaction for play with enriching cat toys for 20–30 min a day. Room temperature was maintained at 22°C with relative humidity at 50 to 60 %. Weekly disinfection with chlorhexidine (Nolvasan; Pfizer) disinfectant and daily cleaning occurred.

### Study design

The experiment employed a randomised complete block design with the cat as a blocking factor. There were three dietary treatments consisting of cornstarch (CHO), poultry fat (FAT) and whey protein (PRO). The calorimetry respiration chambers (Qubit Systems Inc.) were made of Plexiglass with dimensions of 53·3 × 53·3 × 76·2 cm. Cats were previously acclimated to temporary restriction for other studies and procedures^(7,13)^. Each chamber contained food and water bowls, litter box, toys and a fleece bed, with sufficient space between elimination and resting areas. The fleeces, toys, litter and litter box were cleaned and replaced after every 24 h collection period.

On calorimetry days, the cats were placed in individual respiration chambers. After 0·5 h of gas equilibration, two fasting respiration measurements 0·5 h apart were taken for calculating resting VCO_2_ and VO_2_. Once the second fasting measurement was completed, the cats were taken out of their chambers, fed their assigned single macronutrient. The treatments fed were solutions of 22–24 g of poultry fat in a single dose (FAT), 54–56 g of cornstarch mixed in equal amounts of water and provided in two doses (CHO) or a single dose of 56–62 g of whey protein mixed in equal amounts of water (PRO). Kemin^®^ sourced poultry fat (8·6 kcal/g), Kroger^®^ brand cornstarch (3·8 kcal/g) and Lactalis Ingredients^®^ whey protein concentrate (3·4 kcal/g) were used to make the treatments. Poultry fat and cornstarch only had reported crude fat and carbohydrate within the guaranteed analysis. Whey concentrate contained 75·3 % of crude protein, 4·2 % crude fat, 4·1 % ash, 4·8 % moisture and 11·6 % nitrogen-free extract. Briefly, the single macronutrient delivery was provided on an isocaloric basis where males = 210 kcal metabolisable energy/kg BW per day and females = 193 kcal metabolisable energy/kg BW per day based on historical records using modified Atwater equation^(16)^.

The cats were not previously adapted to the treatments. To avoid olfaction-based food selectivity and limited voluntary intake of carbohydrates^(17,18)^, treatments were administered using a 35 ml catheter-tipped syringe, with solutions injected at a voluntary rate directly into the buccal pouch, similar to bottle feeding. The complete volume of the syringe was administered within 5 minutes for all treatments and all cats, after which cats were returned to the chambers for post-meal feeding measures. The duration between each successive measurement was three seconds, and each cat had a total dwell time of 5 minutes to record their gaseous exchange. Respiration calorimetry was done for 24 h after feeding, with recalibration of O_2_ and CO_2_ background reference being conducted every 7 to 8 h. Once complete, the cats were returned into their free-living room.

### Calculations and statistics

All cats had their O_2_, and CO_2_ gaseous exchange recorded automatically on Qubit software every 5 min. The RQ and EE were calculated using the following equations:

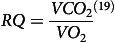




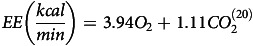




The EE and RQ values were averaged for analysis in two different ways. First, the mean EE and RQ of FAT, CHO and PRO treatments were obtained from the total 0 to 24 h calorimetry period and considered the average daily value. The second set of means for EE and RQ was calculated from the 2 h fasting period and each 4 h time increment post-feeding of the 24 h calorimetry period. The latter included comparison of means from 0 to 4 h, 4 to 8 h, 8 to 12 h, 12 to 16 h, 16 to 20 h, and 20 to 24 h. Area under the curve (AUC) values were also determined for each time increment for EE^(21)^.

All statistical analyses were conducted in SAS software version 9.4 (SAS Institute Inc.) using PROC GLIMMIX. The model used for average values was 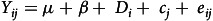 , where 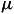 is the intercept,*Y*_*ij*_ is the dependent variable, *D*_*i*_ is the fixed effect of *i* ^th^ treatment used, *c*_*j*_ is the random effect of *j* ^th^ cat, 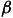 is the covariate and *e*_*ij*_ is the residual error. Repeated measures analysis was performed using: 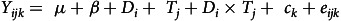 , where *T*_*j*_ is either the 2 h before feeding fasted state or the 4 h time intervals after feeding within a calorimetry period, *D*_*i*_ 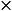 *T*_*j*_ is the interaction between diet and time interval in fasted and post-feeding states and all other variables are as previously described. For the second model, time was considered fixed, and the intra-animal variation was modelled using a first-order autoregressive covariance structure. Fasted values were included as covariates in both models. Differences in diets were compared with the Tukey–Kramer comparison procedure with Kenward–Roger correction. The 4 h increments were compared for significance using the SLICEDIFFS option. Additionally, AUC values were analysed for EE variables using the linear trapezoidal rule where the difference in AUC was estimated to be the difference in postprandial EE and resting fed metabolic rate^(22)^. Resting fed metabolic rate was the lowest observed EE value^(16)^ over the collection period used for these calculations instead of fasted EE, which was higher prior to feeding in the cats used within this study. Furthermore, HIF values were estimated using the AUC of total EE values for each dietary treatment as outlined by Asaro *et al.* ^(23)^. Similar repeated measures analysis was applied for incremental 4 h heat increment of feeding estimates from postprandial TEE. Normality of residuals of the models was assessed using the Shapiro–Wilk test. *P* values less than 0·05 were considered significant, and 0·05 < *P* < 0·10 was identified as a trend.

## Results

Cats fed CHO consistently were observed to excrete white feces after the calorimetry period. Total HIF produced over the 24 h calorimetry period and 4 h postprandial increments is summarised in Table 1. No differences were observed in 24 h HIF values among treatments (Table 1; *P* = 0·26).

**Table 1.** Heat increment of feeding (HIF) estimated from total EE measured in cats (*n* 10) for the 24 h calorimetry period and at 4 h intervals after feeding single macronutrient treatments. Mean estimates are provided with standard error of difference (SED)

Time interval, h	CHO, kcal/100g DM	FAT, kcal/100g DM	PRO, kcal/100g DM	SED	Diet effect *P* ^†^	Diet × Time effect *P* ^‡^
Total	33·3	34·7	41·1	4·86	0·26	–
0–4	5·10^b^	9·66^a^	9·10^ab^	1·69	0·41	0·007
4–8	4·74^b^	6·66^b^	9·23^a^	1·69		
8–12	4·78	5·57	5·91	1·69		
12–16	6·33	2·68	6·47	1·69		
16–20	5·16	3·89	4·30	1·69		
20–24	7·19	6·29	6·10	1·69		

CHO, carbohydrate treatment; FAT, fat treatment; PRO, protein treatment.

Heat increment of feeding calculated using the linear trapezoidal rule^(20)^.

^ab^Mean area under the curve values of energy expenditure with different superscript letters across rows were significantly different.

† *P* value of fixed effects of treatment on heat increment of feeding values for total and at 4 h postprandial time intervals.

‡ *P* value of fixed effects interactions between treatments at 4 h postprandial time intervals for heat increment of feeding.

**Figure 1.**
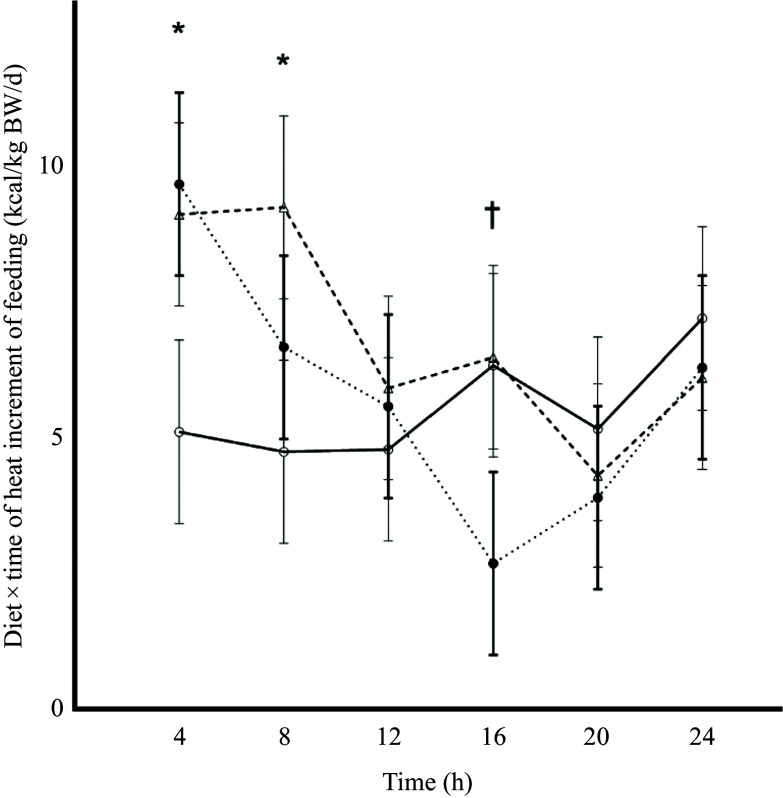
Heat increment of feeding (HIF) values obtained from incremental 4 h area under the curve (AUC) of postprandial total energy expenditure in cats (*n* 10) fed single macronutrient treatments of either a meal of carbohydrate (○, solid line), fat (●, dotted line) or protein (∆, dashed line). Standard error bars represent standard error of mean. * indicates *P* value < 0·05. † represents 0·05 < *P* value < 0·10.

Although the 4 h HIF among diets was similar (*P* = 0·41), there was an interaction (*P* = 0·007; Table 1) among treatments and 4 h time increments that have been summarised within Figure 1. From 0 to 4 h HIF was greater in cats fed FAT as compared with cats fed CHO, while cats fed PRO were similar to both FAT and CHO. From 4 to 8 h, HIF was greater in cats fed PRO than in cats fed either FAT or CHO. The interaction of HIF observed in FAT tended to be lower at 12 to 16 h compared with PRO and CHO (Figure 1; *P* = 0·08).

### Fasted energy expenditure and respiratory quotient

**Figure 2.**
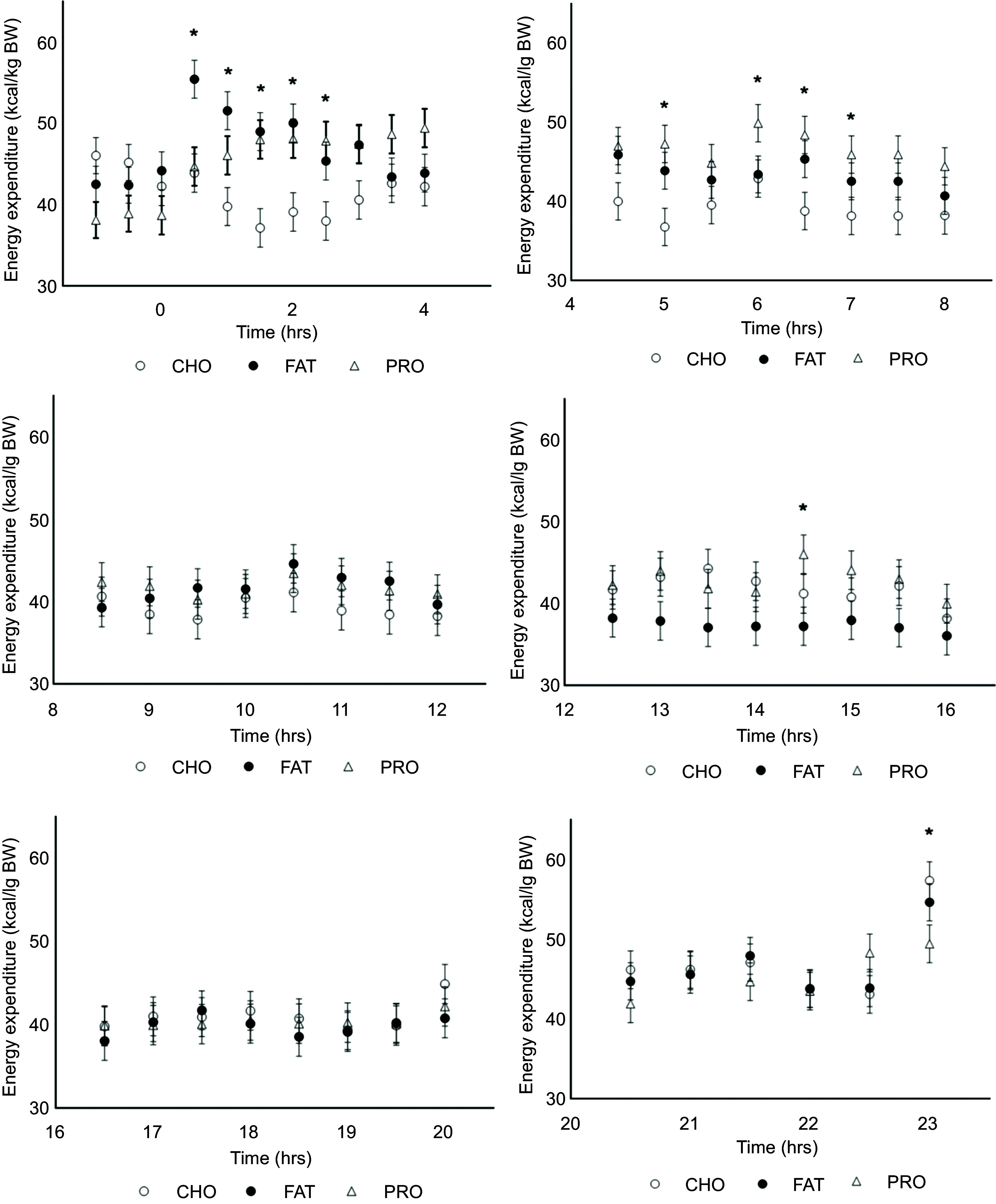
Energy expenditure (EE; kcal/kg BW) and 0·5 h time interactions in cats (*n* 10) fed single macronutrient treatments of either a meal of carbohydrate (○, CHO), fat (●, FAT) or protein (∆, PRO). * indicates *P* value < 0·05.

**Figure 3.**
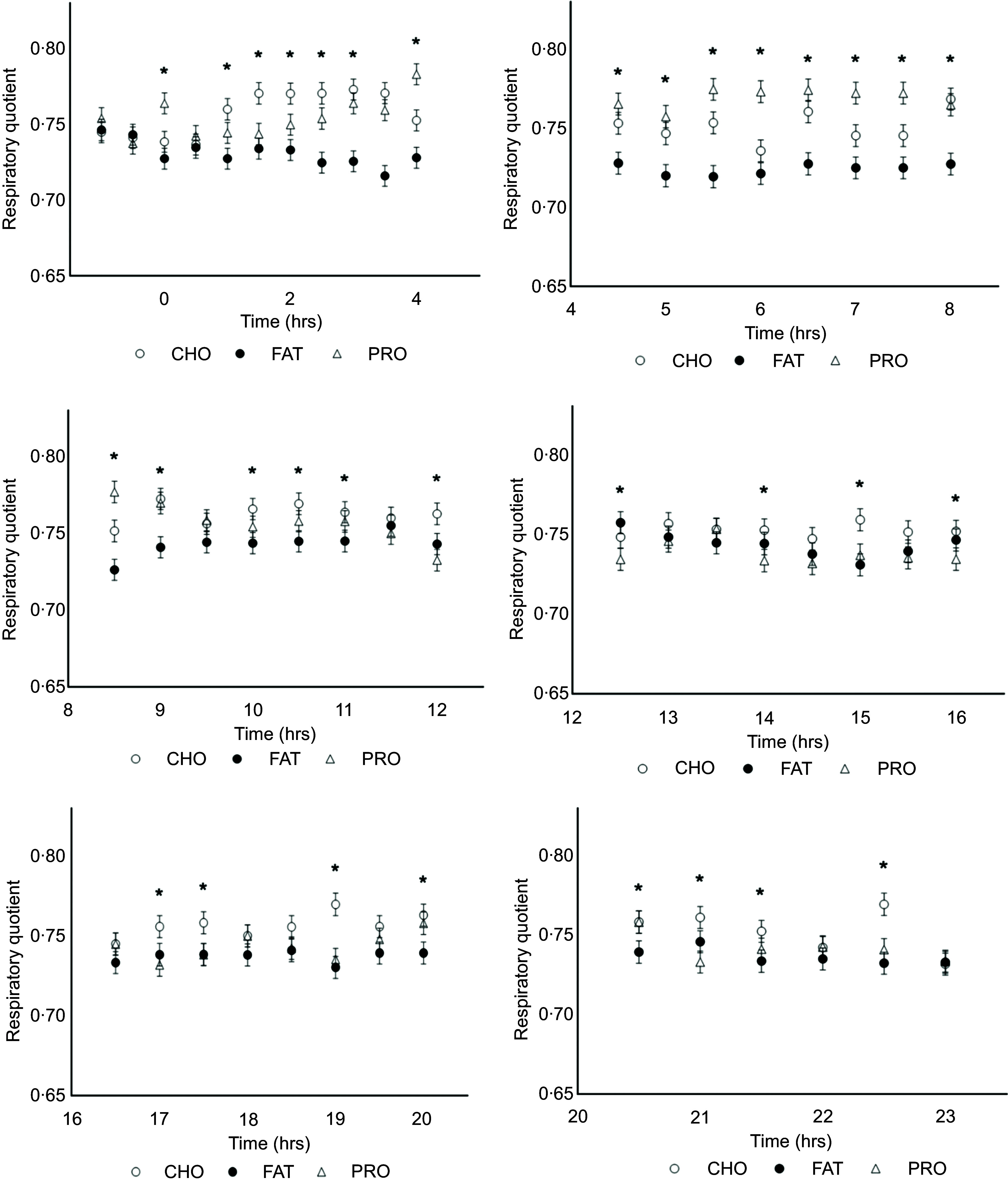
Respiratory quotient and 0·5 h time interactions in cats (*n* 10) fed single macronutrient treatments of either a meal of carbohydrate (○, CHO), fat (●, FAT) or protein (∆, PRO). * indicates *P* value < 0·05.

Fasted EE (Figure 2; Table 2; *P* = 0·73) and RQ (Figure 3; Table 3; *P* = 0·48) were similar among diets as expected since this was prior to treatment delivery. There were no interactions among diet and time during the fasted intervals for EE (Table 2; *P* = 0·77) and RQ (Table 3; *P* = 0·15).

**Table 2.** Energy expenditure (EE) of cats (*n* 10) measured over the total 24 h period, 2 h before feeding (fasted) and at 4 h intervals postprandially when fed single macronutrient treatments. Mean estimates are provided with standard error of difference (SED)

Time interval, h	CHO, kcal/kg BW/d	FAT, kcal/kg BW/d	PRO, kcal/kg BW/d	SED	Diet effect *P* ^†^	Diet × Time effect *P* ^‡^
Total	41·3	42·7	44·0	1·30	0·16	–
Fasted^*^	45·6	42·5	38·5	2·15	0·73	0·77
0–4	40·4^b^	46·4^a^	45·2^a^	2·03	0·16	< 0·001
4–8	40·0^b^	45·0^a^	46·2^a^	2·00		
8–12	40·5	42·5	43·5	2·00		
12–16	42·1	39·7	43·4	2·00		
16–20	40·3	40·8	41·2	2·02		
20–24	47·6	45·0	44·9	2·12		

CHO, carbohydrate treatment; FAT, fat treatment; PRO, protein treatment.

^abc^Different superscripts across rows indicate significant differences within multiple comparisons of energy expenditure means for total 24 h, fasted and 4 h postprandial intervals across dietary treatments.

* Fasted = 2 h before feeding.

† *P* value of fixed effects of treatment on energy expenditure at total 24 h, 2 h fasted and 4 h postprandial time intervals.

‡ *P* value of fixed effects interactions between treatments and 2 h fasted and 4 h postprandial time intervals for energy expenditure.

**Table 3.** Respiratory quotient (RQ) of cats (*n* 10) measured over the total 24 h period, 2 h before feeding (fasted) and at 4 h intervals postprandially when fed single macronutrient treatments. Mean estimates are provided with standard error of difference (SED)

Time interval, h	CHO	FAT	PRO	SED	Diet effect *P* ^†^	Diet × Time effect *P* ^‡^
Total	0·76	0·74	0·75	0·002	< 0·001	–
Fasted^*^	0·74	0·74	0·75	0·006	0·48	0·15
0–4	0·76^a^	0·73^c^	0·75^b^	0·004	< 0·001	< 0·001
4–8	0·75^b^	0·73^c^	0·77^a^	0·004		
8–12	0·76^a^	0·74^b^	0·76^a^	0·004		
12–16	0·76^a^	0·74^b^	0·74^b^	0·004		
16–20	0·75^a^	0·74^b^	0·74^b^	0·004		
20–24	0·75^a^	0·74^b^	0·74^ab^	0·004		

CHO, carbohydrate treatment; FAT, fat treatment; PRO, protein treatment.

^abc^Different superscripts across rows indicate significant differences within multiple comparisons of respiratory quotient means at fasted and 4 h postprandial intervals across dietary treatments.

* Fasted = 2 h before feeding.

† *P* value of fixed effects of treatment on respiratory quotient at total 24 h, 2 h fasted and 4 h postprandial time intervals.

‡ *P* value of fixed effects interactions between treatments and 2 h fasted and 4 h postprandial time intervals for respiratory quotient.

### Fed energy expenditure and respiratory quotient

Mean fed EE were similar over the whole calorimetry period and among treatments (*P* = 0·16; Figure 2; Table 2), but interaction effects were found with 4 h (*P* < 0·001; Table 2) and 0·5 h time intervals (*P* < 0·001; Figure 2).

Mean fed RQ among all treatments was different (*P* < 0·001) over the whole calorimetry period with cats fed CHO (0·76; sem 0·002) and PRO (0·75; sem 0·002) having greater RQ as compared with cats fed FAT (0·74; sem 0·002; Table 3; Figure 3). Interactions between treatment and 0·5 h and 4 h time intervals were observed for fed RQ (*P* < 0·001; Table 3; Figure 3).

### Energy expenditure and time interactions

Throughout the 0 to 4 h time period, cats consuming FAT (46·4 kcal/kg BW/d; sem 1·48) and PRO (45·2 kcal/kg BW/d; sem 1·52) had greater EE estimates than those of cats fed CHO (40·4 kcal/kg BW/d; sem 1·39; *P* < 0·001). The AUC for EE estimates was greatest for FAT and lowest for CHO, with PRO being intermediate and similar to both FAT and CHO (online Supplementary Table 3; *P* = 0·01). When the interactions between diet and 0·5 h time intervals were evaluated, EE remained greater (*P* < 0·001) when cats were fed FAT compared with CHO from 0·5 to 2 h after feeding, but FAT was not greater than PRO after 0·5 to 1 h; PRO was greater than CHO between 1·5 and 2·5 h (online Supplementary Table 1).

During the next 4 to 8 h post-meal feeding (the post-absorptive state), EE of cats fed PRO (46·2 kcal/kg BW/d; sem 1·50) and FAT (45·0 kcal/kg BW/d; sem 1·46) were greater than CHO (40·0 kcal/kg BW/d; sem 1·49; Table 2: Figure 2; *P* < 0·001). PRO AUC for EE estimates was greater compared with CHO (*P* = 0·01), while FAT AUC for EE did not differ in comparison (online Supplementary Table 3). Within this period, the interaction of PRO and EE was greater than CHO at timepoints 5 and from 6·5 to 7·5 h (online Supplementary Table 1; *P* < 0·001).

No differences in EE were found between treatment and time interactions for 4 h intervals 8 to 12 h, 12 to 16 h, 16 to 20 h and 20 to 24 h (Figure 2; Table 2). Similar results were observed for interactions of EE AUC with 4 h time intervals (online Supplementary Table 3). There were a few more significant 0·5 h interval interactions observed for EE among single macronutrient treatments. At 14·5 h, PRO was greater than FAT (online Supplementary Table 1; *P* < 0·001). Within the final timepoint 23 h, feeding the CHO treatment resulted in a greater EE than FAT and PRO (online Supplementary Table 1; *P* < 0·001).

### Respiratory quotient and time interactions

In 0 to 4 h interval, cats fed CHO (0·76; sem 0·003) and PRO (0·75; sem 0·003) had greater (*P* < 0·001) mean RQ than those fed FAT (0·73; sem 0·003; Figure 3; Table 3). Throughout this period, RQ was greater in cats fed CHO than FAT at 1 h (online Supplementary Table 2; *P* < 0·001). The respiratory quotient for CHO remained greater than PRO until 2 h, and at 4 h PRO RQ was greater than CHO. At 4 to 8 h, cats fed PRO (0·77; sem 0·003) had greater mean RQ than those fed CHO (0·75; sem 0·003), and both had greater RQ than those fed FAT (0·73; sem 0·003; Figure 3; Table 3). PRO and CHO RQ remained greater than FAT from 5·5 to 6 h and at 7·5 h (online Supplementary Table 2).

During the 8 to 12 h interval, cats fed CHO (0·76; sem 0·003) had similar RQ to those fed PRO (0·76; sem 0·003), and both had higher RQ than those fed FAT (0·74; sem 0·003; Table 3; *P* < 0·001). Initially, RQ were different for all treatments at 8·5 h (Figure 3; online Supplementary Table 2). However, the FAT RQ increased for the rest of this period. CHO RQ remained higher than fat until 11 h, after which FAT values decreased and remained lower until the end of this period (Figure 3; online Supplementary Table 2). Subsequently, at 12 to 16 h interval cats fed CHO (0·76; sem 0·003) had higher mean RQ compared with cats fed FAT (0·74; sem 0·003) and PRO (0·74; sem 0·003; Figure 3; Table 3; *P* < 0·001). The RQ of FAT was greater than that of PRO at 12·5 h, while the CHO RQ was greater than that of FAT at 15 h. CHO remained greater than PRO from 14 to 16 h (online Supplementary Table 2; *P* < 0·001).

Within 16 to 20 h, cats fed CHO (0·75; sem 0·003) had a higher RQ compared with those fed PRO (0·74; sem 0·003) and FAT (0·74; sem 0·003; Table 3; *P* < 0·001). CHO RQ was greater than PRO at 17 and 19 h, while CHO RQ was higher than FAT at 19 and 20 h (online Supplementary Table 2). Protein RQ increased over fat at the end of this period (online Supplementary Table 2; *P* < 0·001). During the last interval of 20 to 24 h, cats consuming CHO (0·75; sem 0·003; *P* < 0·001) had greater RQ compared with cats fed PRO (0·74; sem 0·003) and FAT (0·74; sem 0·003), both of which were similar (Table 3). CHO RQ rose over FAT and PRO at 20·5 to 21·5 h (online Supplementary Table 2). CHO RQ increased again at 22·5 h over both PRO and FAT within this time interval (online Supplementary Table 2; *P* < 0·001).

## Discussion

To the best of our knowledge, this is the first study assessing how a carnivorous species, domestic cats, acutely respond to being fed single macronutrients or enriched single ingredients. Ingestion of PRO resulted in the greatest EE estimate in the postprandial period and HIF in contrast to consuming CHO or FAT, which agrees with previous data^(11,24)^. The RQ in cats fed a single PRO treatment within the current study was lower than observed in cats fed mixed diets containing different dietary protein concentrations^(2,25)^. Mixed diets contained higher carbohydrates that would result in greater glucose oxidation, leading to a higher reported RQ in the literature^(25)^ which likely accounts for the different RQ within the current study. The PRO diet used in the present study only allowed cats to utilise glucose available from gluconeogenesis using dietary derived AA, consequently decreasing RQ^(10)^.

In comparison with PRO, FAT had an immediate peak within the acute postprandial phase at 4 h, simultaneously lowering RQ likely due to the increase in plasma fatty acids and triglycerides^(26)^. Lower EE was observed in cats fed FAT as single macronutrients, compared to literature where mixed diets with different fat concentrations were provided^(1,7)^; however, the acute response to being fed greater fat remained the same as previous studies. Fat contributes to hepatic gluconeogenesis through the incorporation of carbon atoms from glycerol^(27)^. Cats utilise dietary glycerol, which aligns with the higher EE observed for the FAT diet in the current study^(27)^. This emphasises that cats are more flexible in utilising dietary fat to produce glucose for metabolic functions compared with omnivorous species. FAT resulted in the lowest RQ and is expected since the RQ for fat oxidation is approximately 0·7^(6,28)^. Similar to Lester *et al.* ^(1)^, RQ in the current study was found to be lower 8 h post feeding in cats fed FAT. The rapid decline in RQ postprandially indicates that cats did not have delayed gastric emptying when fed a single poultry fat dose, which contrasts with reported results in humans^(29,30)^. This suggests that fat oxidation and gluconeogenesis are the dominant methods of energy production during this period.

Additionally, the natural prey diet of cats contains high protein and fat; therefore, higher rates of gluconeogenesis would be expected for obligate carnivores. In contrast, omnivores naturally consume higher proportions of dietary starch and have greater capacity to ferment dietary fibre and use short-chain fatty acids for energy production depending on species-specific adaptations^(31)^. When comparing EE changes in 24 h in other studies, cats increase fat oxidation in response to greater fat intake as an adaptation to meat-based diets^(1)^ and cats peaked in carbohydrate oxidation faster than fat^(7)^; however, this could stem from the use of diets containing carbohydrates. In contrast, the FAT used for the treatment in this study had no carbohydrate, and we expect it to be highly digestible. The RQ of cats fed FAT rises and falls within the 8–15 h duration and remains lower for the rest of the total period, indicating that the low availability of fat caused the switch to alternate energy sources, such as protein produced from the degradation of intestinal epithelium and subsequent mobilisation of lipids from adipocytes, for free fatty acids^(28)^.

Digestibility was not measured as part of this study; however, the presence of white feces in cats fed CHO indicates lower digestibility of the cornstarch treatment, evidenced by the lowest EE produced overall. In contrast, PRO treatment had greater glucose utilisation from gluconeogenesis^(9)^ evidenced by the rise in EE and RQ at 4–7 h in comparison to CHO. Deng *et al.* ^(32)^ observed cats fed maltodextrin had glucose remain higher 6 h after feeding compared with high-protein diet, which indicates that cats are capable of utilising glucose and thereby EE when fed a bioavailable carbohydrate source. However, within the present study, the lowest EE in cats fed CHO contrasts previous research where cats were fed varying dietary carbohydrates^(2,8,11)^ reported higher EE. This low EE differs from the acute increase in EE observed in humans when glucose is oxidised to generate ATP^(33)^ and suggests that cats are better at gluconeogenesis using AA precursors than they are at the digestion, absorption and tissue utilisation of glucose derived from dietary starch^(9)^. Additionally, cats have lower amylase activity^(34)^, reduced rates of Na-dependent glucose uptake in small intestinal brush border^(35)^, lower mRNA expression for enzymes involved in starch digestion than omnivores^(36)^ and lack hepatic glucokinase, which limits glucose oxidation^(37)^. The lower EE observed from CHO treatment used in this study likely stems from cats having a lower peak in plasma glucose concentration when fed a high-carbohydrate meal, resulting from a combination of delayed gastric emptying when compared with FAT, lower activity of key digestive enzymes and lower glucose oxidation. This suggests that carbohydrates may limit energy expenditure when fed alone in cats^(38)^. Previous geometric analyses have shown that cats have a ‘carbohydrate ceiling’ of 72 kcal per day within their extruded food intake^(38)^. Interestingly, all three treatments displayed a spike in EE at 20–24 h, which captures the crepuscular nature of activity within cats^(39)^.

The respiratory quotient of cats fed CHO was lower but remained within the expected range found in cats fed mixed diets and more specifically, between 0·74 and 0·86^(1,2,11,40–42)^. Omnivorous species, such as humans, dogs and chickens, have an RQ of approximately 1·0 when carbohydrates are fed as a single macronutrient^(3,6,43,44)^, suggesting glycogen and lipid deposition^(45)^. An RQ less than 1·0 suggests that even when carbohydrates are the only source of dietary energy, cats cannot completely oxidise this substrate in a short period of time and continually synthesise glucose from amino acid precursors. Gluconeogenesis is equally important for carnivores, herbivores and omnivores in fasted states^(46)^. Nevertheless, Eisert^(9)^ suggested that glucose-requiring feline brain tissues depend on AA catabolism to compensate for natural prey diets, which are composed of low carbohydrate and high protein^(47)^. The resulting lower RQ when cats are fed PRO compared with CHO suggests that they are mainly producing and utilising glucose for energy from AA through gluconeogenesis^(28)^. CHO treatment increased the RQ within the first 2 h, similar to what has been observed in previous studies^(32,41,42)^. The fluctuations in PRO and CHO diet RQ indicate continued breakdown of amino acids produced through gluconeogenesis until the end of the calorimetry period.

The current study quantified the postprandial response or HIF for 24 h, similar to Asaro *et al.* ^(23)^, potentially explaining why the values are greater in comparison with previous thermogenic response studies that measured HIF at 7–14 h post-meal consumption^(24,48)^. Cats fed PRO had the greatest HIF compared with FAT and CHO, which is expected as PRO turnover is an energy-demanding physiological process^(24,48,49)^ and plays a greater role in carnivores compared with omnivorous or herbivorous species^(49–53)^. The HIF responds to both the composition of the diet and the energy and food intake of individual animals^(53)^. PRO and FAT treatments resulted in greater peaks in thermogenesis within the first few hours of feeding. Heat production or EE remained greater in humans fed PRO enriched treatments for the first 4 h in comparison to FAT and CHO^(24)^. Interestingly, single FAT treatment also demonstrated a similar thermogenic peak for cats in this study within the acute post-feeding phase as observed in humans. The peak subsequently dropped after 4 h; however, this suggests improved efficiency within carnivores to utilise larger amounts of fat for digestion and absorption processes from natural prey, in contrast to omnivorous species. The tendency of declining HIF for cats fed FAT at 16 h coincides with the FAT RQ value of 0·75, a midpoint between CHO and PRO. This requires further investigation to determine whether this is an indicator of the lack of substrates from FAT being available for thermogenesis within an elongated postprandial period.

The present study attempted to supply equal amounts of dietary energy from CHO, FAT and PRO using the modified Atwater calculation to predict dietary energy density, but digestible or metabolisable energy of these ingredients or efficiency of utilisation was not measured. Furthermore, data on metabolites and hormones in the extracellular compartment would support the interpretation of differences in intermediary metabolism and macronutrient partitioning; however, we chose not to sample blood due to ethical constraints. Measuring digestibility of cooked *v*. raw cornstarch as a treatment and using alternative carbohydrate sources that are easily digested may improve our understanding of the acute responses in RQ and EE observed. Additionally, the efficiency of absorption and utilisation of single nutrient-enriched treatments could be addressed through more research to provide insight on the differences in flexibility of macronutrient use observed within this study.

In conclusion, when single macronutrients were fed, RQ and EE responded as hypothesised. However, when compared with other omnivorous species, the values were lower, likely due to limited metabolic flexibility of obligate carnivores and with consistent reliance on gluconeogenesis. HIF remained similar overall across single macronutrient treatments. Since cats were fed the macronutrients alone, they could not rely on other nutrients to support EE, and we observed lower EE than when cats were fed mixed diets^(2,7)^. These results support previous research suggesting that carnivores lack metabolic flexibility and handle dietary carbohydrates differently.

## Supporting information

Jantzi et al. supplementary material Jantzi et al. supplementary material

## Supplementary material

For supplementary material/s referred to in this article, please visit https://doi.org/10.1017/S0007114525105850
