# Supplementary material for: Macronutrient and energy metabolism changes in domestic cats when fed cornstarch, whey protein, and, poultry fat
Source: Br J Nutr. 2025 Dec 10;135(3):241–9. doi: 10.1017/S0007114525105850 (PMC12912840; doi:10.1017/S0007114525105850)
Supplement: Jantzi et al. supplementary material [file S0007114525105850sup001.docx]

**Supplement**

Table 1: Mean energy expenditure and 0.5 h time interval interactions in cats fed a meal of either carbohydrate, protein or fat (Diet x Time *P* < 0.001).

| Time, h | CHO, kcal/kg BW/d | FAT, kcal/kg BW/d | PRO, kcal/kg BW/d | SED |
| --- | --- | --- | --- | --- |
| -1 | 46.1 | 42.5 | 38.1 | 2.15 |
| -0.5 | 45.2 | 42,4 | 38.9 | 2.15 |
| 0 | 42.3 | 44.2 | 38.7 | 3.27 |
| 0.5 | 43.9^b^ | 55.5^a^ | 44.7^b^ | 3.27 |
| 1 | 39.8^b^ | 51.6^a^ | 46.1^ab^ | 3.27 |
| 1.5 | 37.2^b^ | 50.0^a^ | 48.0^a^ | 3.27 |
| 2 | 39.1^b^ | 50.1^a^ | 48.1^a^ | 3.27 |
| 2.5 | 38.0^b^ | 45.4^ab^ | 47.9^a^ | 3.27 |
| 3 | 40.6 | 47.4 | 47.5 | 3.27 |
| 3.5 | 42.6 | 43.4 | 48.7 | 3.27 |
| 4 | 42.2 | 43.9 | 49.5 | 3.27 |
| 4.5 | 40.0 | 45.9 | 47.0 | 3.27 |
| 5 | 36.8^b^ | 43.9^ab^ | 47.3^a^ | 3.27 |
| 5.5 | 39.6 | 42.7 | 44.9 | 3.27 |
| 6 | 42.9 | 43.4 | 49.9 | 3.27 |
| 6.5 | 38.8^b^ | 45.4^ab^ | 48.4^a^ | 3.27 |
| 7 | 38.2^b^ | 42.6^ab^ | 45.9^a^ | 3.27 |
| 7.5 | 38.2^b^ | 42.6^ab^ | 45.9^a^ | 3.27 |
| 8 | 38.2 | 40.7 | 44.4 | 3.27 |
| 8.5 | 40.6 | 39.3 | 42.4 | 3.27 |
| 9 | 38.4 | 40.4 | 41.9 | 3.27 |
| 9.5 | 37.8 | 41.6 | 40.2 | 3.27 |
| 10 | 40.4 | 41.5 | 40.9 | 3.27 |
| 10.5 | 41.1 | 44.6 | 43.4 | 3.27 |
| 11 | 38.9 | 42.9 | 42.0 | 3.27 |
| 11.5 | 38.4 | 42.5 | 41.3 | 3.27 |
| 12 | 38.2 | 39.6 | 40.9 | 3.27 |
| 12.5 | 41.7 | 38.3 | 42.3 | 3.27 |
| 13 | 43.2 | 37.9 | 44.0 | 3.27 |
| 13.5 | 44.3 | 37.1 | 41.8 | 3.27 |
| 14 | 42.7 | 37.3 | 41.4 | 3.27 |
| 14.5 | 41.2^ab^ | 37.2^b^ | 46.0^a^ | 3.27 |
| 15 | 40.8 | 38.0 | 44.1 | 3.27 |
| 15.5 | 42.2 | 37.1 | 43.0 | 3.27 |
| 16 | 38.2 | 36.1 | 40.0 | 3.27 |
| 16.5 | 39.9 | 38.1 | 39.9 | 3.27 |
| 17 | 41.1 | 40.3 | 40.0 | 3.27 |
| 17.5 | 40.9 | 41.8 | 40.1 | 3.27 |
| 18 | 41.7 | 40.2 | 40.5 | 3.27 |
| 18.5 | 40.8 | 38.6 | 40.2 | 3.27 |
| 19 | 39.4 | 39.2 | 40.3 | 3.27 |
| 19.5 | 39.9 | 40.2 | 40.2 | 3.27 |
| 20 | 44.9 | 40.8 | 42.2 | 3.27 |
| 20.5 | 46.2 | 44.7 | 41.9 | 3.27 |
| 21 | 46.2 | 45.6 | 46.1 | 3.27 |
| 21.5 | 47.1 | 47.9 | 44.7 | 3.27 |
| 22 | 43.8 | 43.8 | 43.5 | 3.27 |
| 22.5 | 43.1 | 43.9 | 48.3 | 3.27 |
| 23 | 57.4^a^ | 54.7^ab^ | 49.4^b^ | 3.27 |

^ab^Different superscripts across rows indicate significant differences within the multiple comparisons of energy expenditure and time interactions
CHO: Carbohydrate treatment; FAT: Fat treatment; PRO: Protein treatment

Table 2: Mean respiratory quotient and 0.5 h time interval interactions in cats fed a meal of either carbohydrate, protein or fat (Diet x Time *P* < 0.001)

| Time, h | CHO | FAT | PRO | SED |
| --- | --- | --- | --- | --- |
| -1 | 0.74 | 0.75 | 0.75 | 0.007 |
| -0.5 | 0.74 | 0.74 | 0.74 | 0.007 |
| 0 | 0.74^b^ | 0.73^b^ | 0.76^a^ | 0.007 |
| 0.5 | 0.74 | 0.73 | 0.74 | 0.007 |
| 1 | 0.76^a^ | 0.73^b^ | 0.74^a^ | 0.007 |
| 1.5 | 0.77^a^ | 0.73^b^ | 0.74^b^ | 0.007 |
| 2 | 0.77^a^ | 0.73^b^ | 0.75^c^ | 0.007 |
| 2.5 | 0.77^a^ | 0.72^b^ | 0.75^c^ | 0.007 |
| 3 | 0.77^a^ | 0.73^b^ | 0.76^a^ | 0.007 |
| 3.5 | 0.77 | 0.72 | 0.76 | 0.007 |
| 4 | 0.75^b^ | 0.73^c^ | 0.78^a^ | 0.007 |
| 4.5 | 0.75^b^ | 0.73^b^ | 0.77^a^ | 0.007 |
| 5 | 0.75^a^ | 0.72^b^ | 0.76^a^ | 0.007 |
| 5.5 | 0.75^b^ | 0.72^c^ | 0.77^a^ | 0.007 |
| 6 | 0.74^b^ | 0.72^b^ | 0.77^a^ | 0.007 |
| 6.5 | 0.76^a^ | 0.73^b^ | 0.77^a^ | 0.007 |
| 7 | 0.75^b^ | 0.73^c^ | 0.77^a^ | 0.007 |
| 7.5 | 0.75^b^ | 0.73^c^ | 0.77^a^ | 0.007 |
| 8 | 0.77^a^ | 0.73^b^ | 0.76^a^ | 0.007 |
| 8.5 | 0.75^b^ | 0.73^c^ | 0.78^a^ | 0.007 |
| 9 | 0.77^a^ | 0.74^b^ | 0.77^a^ | 0.007 |
| 9.5 | 0.76 | 0.74 | 0.76 | 0.007 |
| 10 | 0.77^a^ | 0.74^b^ | 0.75^ab^ | 0.007 |
| 10.5 | 0.77^a^ | 0.74^b^ | 0.76^ab^ | 0.007 |
| 11 | 0.76^a^ | 0.75^b^ | 0.76^ab^ | 0.007 |
| 11.5 | 0.76 | 0.76 | 0.75 | 0.007 |
| 12 | 0.76^a^ | 0.74^b^ | 0.73^b^ | 0.007 |
| 12.5 | 0.75^ab^ | 0.76^a^ | 0.73^b^ | 0.007 |
| 13 | 0.76 | 0.75 | 0.75 | 0.007 |
| 13.5 | 0.75 | 0.74 | 0.75 | 0.007 |
| 14 | 0.75^a^ | 0.74^ab^ | 0.73^b^ | 0.007 |
| 14.5 | 0.75 | 0.74 | 0.73 | 0.007 |
| 15 | 0.76^a^ | 0.73^b^ | 0.74^b^ | 0.007 |
| 15.5 | 0.75 | 0.74 | 0.74 | 0.007 |
| 16 | 0.75^a^ | 0.75^ab^ | 0.73^b^ | 0.007 |
| 16.5 | 0.75 | 0.73 | 0.75 | 0.007 |
| 17 | 0.76^a^ | 0.74^b^ | 0.73^b^ | 0.007 |
| 17.5 | 0.76^a^ | 0.74^b^ | 0.74^b^ | 0.007 |
| 18 | 0.75 | 0.74 | 0.75 | 0.007 |
| 18.5 | 0.76 | 0.74 | 0.74 | 0.007 |
| 19 | 0.77^a^ | 0.73^b^ | 0.74^b^ | 0.007 |
| 19.5 | 0.76 | 0.74 | 0.75 | 0.007 |
| 20 | 0.76^a^ | 0.74^b^ | 0.76^a^ | 0.007 |
| 20.5 | 0.76^a^ | 0.74^b^ | 0.76^a^ | 0.007 |
| 21 | 0.76^a^ | 0.75^ab^ | 0.73^b^ | 0.007 |
| 21.5 | 0.75^a^ | 0.73^b^ | 0.74^ab^ | 0.007 |
| 22 | 0.74 | 0.74 | 0.74 | 0.007 |
| 22.5 | 0.77^a^ | 0.73^b^ | 0.74^b^ | 0.007 |
| 23 | 0.73 | 0.73 | 0.73 | 0.007 |

^abc^Differemt superscripts across rows indicate significant differences within multiple comparisons of respiratory quotient and time interactions

CHO: Carbohydrate treatment; FAT: Fat treatment; PRO: Protein treatment

Table 3: Mean area under the curve values for energy expenditure interactions at 4 h post feeding intervals for cats fed single meal of CHO, FAT or PRO

| Time interval, h | CHO, kcal/kg BW/d | FAT, kcal/kg BW/d | PRO, kcal/kg BW/d | SED | Diet effect *P*† | Diet x Time effect *P*‡ |
| --- | --- | --- | --- | --- | --- | --- |
| 0 – 4 | 1625^b^ | 3255^a^ | 2820^b^ | 584.40 | 0.33 | 0.01 |
| 4 – 8 | 1788^b^ | 2496^b^ | 3450^a^ | 584.40 |  |  |
| 8 – 12 | 1637 | 1977 | 2210 | 584.40 |  |  |
| 12 – 16 | 2194 | 1127 | 2324 | 584.40 |  |  |
| 16 – 20 | 1912 | 1359 | 1722 | 584.40 |  |  |
| 20 – 24 | 2794 | 2403 | 2384 | 584.40 |  |  |

^ab^Different superscripts across rows indicate significant differences within multiple comparisons of area under the curve estimated from postprandial energy expenditure of dietary treatments

†*P*-value of fixed effects of treatment on area under the curve of energy expenditure at 4 h postprandial time intervals

‡*P*-value of fixed effects interactions between treatments and 4 h postprandial time intervals for area under the curve of energy expenditure

CHO: Carbohydrate treatment; FAT: Fat treatment; PRO: Protein treatment
